# Cutaneous reaction to ifosfamide plus mesna treated with desensitization challenge: a case report

**DOI:** 10.1186/s12948-022-00173-0

**Published:** 2022-05-23

**Authors:** Ana Delgado-Prada, Julián Borrás, Roxana Farzanegan, María Cruz Torres Gorriz, Adrián Germán-Sánchez, Raquel Cervera Aznar, Isabela Raducan, Jose Vicente Castelló, Alfredo Sanchez-Hernandez, Ernesto Enrique

**Affiliations:** 1 Allergy Department, Valencia University Clinical Hospital, Avenida Blasco No. 17, CP 46021 Valencia, Spain; 2 Allergy Department, Provincial University Consortium Hospital, Castellon, Spain; 3 Allergy Department, Castellon University General Hospital, Castellon, Spain; 4 Oncology Department, Provincial University Consortium Hospital, Castellon, Spain

**Keywords:** Ifosfamide plus mesna, Desensitization, Chemotherapy

## Abstract

**Background:**

Ifosfamide is an alkylating agent used in the treatment of a wide range of tumours. Because of known side effects it is usually administered in combination with mesna, a thiol agent with uroprotective activity, to reduce them and increase the therapeutic dose. The most frequently administered regimens for ifosfamide are fractionated doses for 3 to 5 days, high-dose intravenous bolus, and continuous infusion over 24 to 72 h. Hypersensitivity reactions to ifosfamide plus mesna are not frequently described in the literature. Moreover, no reports exist concerning desensitization for this chemotherapy combination.

**Case presentation:**

A 47-year-old man with stage IV renal sarcoma was treated with the combination of ifosfamide and mesna every 3 weeks in a 4-consecutive-day infusion protocol. During the second cycle of chemotherapy, he presented acute cutaneous symptoms. A 12-step desensitization protocol was proposed in view of the lack of knowledge of the possible hypersensitivity reactions to this combination of chemotherapy agents, and the multiple difficulties found during the study of the case.

**Conclusions:**

The 12-step desensitization protocol was well tolerated. Therefore, it is an appropriate and safe option in the case of suspected allergy to ifosfamide plus mesna.

## Background

Ifosfamide is an antineoplastic agent with alkylating activity used in monotherapy or combination chemotherapy. Multiple complications have been described because of its toxicity. The most reported side effects of this drug have included arrhythmias, heart failure, nephrotoxicity, hemorrhagic cystitis, severe encephalopathy, peripheral neuropathy, as well as interaction with other drugs. Toxicity has been related to the need to use high dose of ifosfamide to be effective and the administration method chosen. The most commonly used administration regimes are fractionated doses for 3 to 5 days, high-dose intravenous bolus, and continuous infusion over 24 to 72 h.

To avoid some of these side effects it is usually administered in combination with mesna (2-mercaptoethane sulfonate). The latter is a thiol agent with uroprotective activity that prevents side effects such as hemorrhagic cystitis or nephrotoxicity, allowing the administration of higher doses of ifosfamide without undesirable side effects. Ifosfamide-mesna is administered as a continuous infusion and not as a bolus to avoid central nervous system toxicity. However, mesna has no protective activity over other complications.

This combination therapy can be used in a wide range of tumours, such as testicular, lung, lymphoma, advanced solid, soft tissue sarcomas, and gynecological tumours [1–4].

## Case presentation

We report the case of a 47-year-old male with the diagnosis of stage IV renal sarcoma who received treatment with ifosfamide-mesna every 3 weeks, following a consecutive 4-day infusion protocol in which he received 3 fractionated dosages/bags per day (1 bag every 8 h). During the first day of the second chemotherapy cycle, immediately after completing the infusion of the third bag, the patient began to experience itchy urticarial lesions in the lower abdomen and in the inguinal area, which progressively generalised to the rest of the body. The infusion was stopped, and the reaction was treated with intravenous hydrocortisone and dexchlorpheniramine. Only a slight improvement in the clinical condition was noticed after the treatment. Therefore, chemotherapy regimen was discontinued, and the patient was referred for an allergological study.

The patient´s history included rhinitis and mild allergic asthma to house dust mites and pollens with adequate control. It should be noted that three weeks earlier, during the first cycle, the patient had suffered a similar skin reaction on administration of the last dose of a 4-day treatment regimen. Due to the intense pruritus in the following days after the infusion, medical assistance was required at an Emergency Department. Home ebastine regimen was given and maintained for one week with progressive resolution. There is no graphic documentation of this first reaction, nor an assessment by a dermatologist or allergist. In the current cutaneous reaction, symptomatic treatment with ebastine was maintained with resolution after a few days with no residual lesions.

The clinical picture suggested an acute urticaria caused by a possible hypersensitivity reaction to the combination of ifosfamide and mesna. Several problems were presented during our study. First, there is no evidence on how to perform the skin test with these drugs, in addition we could not do it because of the need to administer the treatment only 2 days after seeing the patient for the first time. Second, the lack of alternative therapies by oncology. Third, the fractionated form of administration over 4 days every 3 weeks. And finally, the rapid progression of the disease.

With all that has been previously described, we considered that the subsequent cycles of chemotherapy could be administered using a standardized 12-step desensitization protocol, as described below [5].

Home and hospital pre-medication were administered together with the oncological pre-medication (Table 1) [5]. Desensitization to the first bag was completed in 12 steps (performed with a minor variation), reaching an infusion rate of 132 ml/h (standard for this treatment) without incidents (Table 1). It should be noted that the protocol adopted is only implemented in the first bag of the 4 days treatment. By this, we emphasize that rapid desensitization does not result in long-term tolerance. Hence, the patient must be desensitized each time they are exposed to the allergenic drug. However, in certain cases such as desensitization to aspirin for daily use or during an antibiotic course in which the antibiotic is given at regular intervals, if the drug is preserved at pharmacological levels by daily administration, the desensitized state is maintained [6].

**Table 1:** 12-Step desensitization protocol with 1695 mg Ifosfamide + 1695 mg Mesna

Domiciliary Allergy premedication (Start 2 days before)	- Singulair 10 mg
	- Aspirine 300 mg
	- Ebastine 10 mg
	- Ranitidine 150 mg
Hospital Oncology premedication	- Dexamathasone 8 mg intravenous
	- Ganisetron 1 mg oral
Hospital Allergy premedication	- Polaramine 1 vial intravenous
	- Ranitidine 50 mg intravenous
	- Diazepam 5 mg sublingual

3 dilutions of the total doses with 1000 ml 0.9% NaCl	Total doses	mg/ml	
Solution 1/100	16.95	0.01695	
Solution 1/10	169.5	0.1695	
Solution 1/1*	1691.665	1.691665	

	Rate (ml/h)	Time (min)	Vol. Infused (ml)	Doses (mg)
Solution 1/100	2	15	0.5	0.008
	5	15	1.25	0.021
	10	15	2.5	0.042
	20	15	5	0.085
	Total =	60	9.25	0.157
Solution 1/10	5	15	1.25	0.212
	10	15	2.5	0.424
	20	15	5	0.848
	40	15	10	1.695
	Total =	60	18.75	3.178
Solution 1/1	10	15	2.5	4.238
	20	15	5	8.475
	40	15	10	16.950
	80	15	20	33.900
	132**	437	960.53	1628.103
	Total =	617	1000	1691.665
			Total mg	1695

*In this solution we take into acount the previous doses administered in Solution 1/100 and 1/10, which are subtracted from this solution

**Variation of the protocol adding a 13 step

For the remaining 4 days of treatment, chemotherapy was administered according to the normal infusion protocol and 1 tablet of montelukast and ebastine were administered daily as a precautionary measure. On the third day of this first desensitization cycle, with a normal infusion protocol in several bags, a mild urticarial rash appeared without additional symptomatology. Serum tryptase and interleukin (IL) 6 levels were requested 2 h after the reaction, with values within normal range (Tryptase: 5.4 μg/l and IL-6: 7.2 pg/ml). Therefore, it was decided to increase the ebastine regimen to 2 tablets daily.

The clinical picture was resolved with the modified premedication without further complications until the end of the whole cycle.

In succeeding desensitizations cycles, the regimen of montelukast daily and ebastine every 12 h for 5 days was maintained. This was associated with intravenous dexamethasone (4 mg every 8 h), which was decreased to every 12 h on the fourth day, and every 24 h on the last day. Five more cycles were later completed following this same schedule, without further complications.

## Discussion and conclusions

Here we present the case of an urticarial rash in an oncological patient, associated with the administration of ifosfamide plus mesna. Due to several difficulties that occurred during the allergological study of the patient, being the main ones the lack of validated skin tests, the impossibility to perform them, and the clinical history presented, we proposed the completion of a 12-step desensitization protocol on the first day of treatment with normal infusion on the next four days. The aim of this treatment was to emphasise the possibility of reintroducing the culprit anti-cancer drugs safely and effectively, despite the difficulties encountered.

The desensitization protocol we used was well tolerated, suggesting its appropriateness in patients with suspected allergy to ifosfamide plus mesna. Even if only the value of one medication is given, the values for each drug would be the same considering them independently. The milligrams per millilitre are twice the indicated values.

## Data Availability

All data are available upon request.
